# Improving the Anti-Tumor Effect of Indoleamine 2,3-Dioxygenase Inhibitor CY1-4 by CY1-4 Nano-Skeleton Drug Delivery System

**DOI:** 10.3390/jfb15120372

**Published:** 2024-12-09

**Authors:** Hui Li, Junwei Liu, Jingru Wang, Zhuoyue Li, Jianming Yu, Xu Huang, Bingchuan Wan, Xiangbao Meng, Xuan Zhang

**Affiliations:** 1 Department of Pharmaceutics, School of Pharmaceutical Sciences, Peking University, Beijing 100191, China; 1811110118@bjmu.edu.cn (H.L.); 2311210048@stu.pku.edu.cn (J.L.); wjrpku@pku.edu.cn (J.W.); lizhuoyue1001@163.com (Z.L.); 2010307327@stu.pku.edu.cn (J.Y.); 2411210069@stu.pku.edu.cn (X.H.); 2110307330@stu.pku.edu.cn (B.W.); 2 State Key Laboratory of Natural and Biomimetic Drugs, School of Pharmaceutical Sciences, Peking University, Beijing 100191, China; 3 Beijing Key Laboratory of Molecular Pharmaceutics and New Drug Delivery Systems, School of Pharmaceutical Sciences, Peking University, Beijing 100191, China; 4 Ningbo Institute of Marine Medicine, Peking University, Ningbo 315832, China

**Keywords:** MSNM@CY1-4, indoleamine 2,3-dioxygenase, nano-skeleton, anti-tumor, anti-tumor immune

## Abstract

**Background:** CY1-4, 9-nitropyridine [2′,3′:4,5] pyrimido [1,2-α] indole -5,11- dione, is an indoleamine 2,3-dioxygenase (IDO) inhibitor and a poorly water-soluble substance. It is very important to increase the solubility of CY1-4 to improve its bioavailability and therapeutic effect. In this study, the mesoporous silica nano-skeleton carrier material Sylysia was selected as the carrier to load CY1-4, and then the CY1-4 nano-skeleton drug delivery system (MSNM@CY1-4) was prepared by coating the hydrophilic polymer material Hydroxypropyl methylcellulose (HPMC) and the lipid material Distearoylphosphatidyl-ethanolamine-poly(ethylene glycol)_2000_ (DSPE-PEG_2000_) to improve the anti-tumor effect of CY1-4. **Methods**: The solubility and dissolution of MSNM@CY1-4 were investigated, and its bioavailability, anti-tumor efficacy, IDO inhibitory ability and immune mechanism were evaluated in vivo. **Results**: CY1-4 was loaded in MSNM@CY1-4 in an amorphous form, and MSNM@CY1-4 could significantly improve the solubility (up to about 200 times) and dissolution rate of CY1-4. In vivo studies showed that the oral bioavailability of CY1-4 in 20 mg/kg MSNM@CY1-4 was about 23.9-fold more than that in 50 mg/kg CY1-4 suspension. In B16F10 tumor-bearing mice, MSNM@CY1-4 significantly inhibited tumor growth, prolonged survival time, significantly inhibited IDO activity in blood and tumor tissues, and reduced Tregs in tumor tissues and tumor-draining lymph nodes to improve anti-tumor efficacy. **Conclusions**: The nano-skeleton drug delivery system (MSNM@CY1-4) constructed in this study is a potential drug delivery platform for improving the anti-tumor effect of oral poorly water-soluble CY1-4.

## 1. Introduction

Indoleamine 2,3-dioxygenase (IDO) is a heme-containing oxidoreductase that is highly expressed in a variety of tumor cells, including melanoma [1,2] and immune cells [3]. It catalyzes the catabolism of the essential amino acid L-tryptophan (Trp) to L-kynurenine (Kyn) [4,5,6,7]. The consumption of Trp and the accumulation of Kyn will inhibit the differentiation and function of effector T cells and promote the proliferation of regulatory T cells, which will help to form an immunosuppressive microenvironment at the tumor site [8,9,10]. Therefore, IDO inhibitors that can alleviate the immunosuppressive tumor microenvironment and trigger the host immune system have broad application prospects in anti-tumor therapy [11,12,13].

At present, there are no IDO inhibitors listed on the market, and a large number of IDO inhibitors are in the pre-clinical research and development stage. According to their chemical structure, they are mainly divided into tryptophan derivatives [14,15], aryl imidazole derivatives [16], N-hydroxy amidines [17], quinoline compounds [18], and so on. IDO inhibitors entering the clinical research stage mainly include Indoximod, Epacadostat, Navoximod, PF-06840003, Linrodostat, LY-3381916, KHK-2455, NLG-802, and RiMO-301 [19,20,21].

Almost all IDO inhibitors entering clinical research are administered orally. IDO inhibitors have been shown to be an effective immune modulator to synergize with other therapies (such as chemotherapy) by reversing the immunosuppressive tumor microenvironment [22,23,24]. However, problems such as poor water solubility [25], large dosage and high frequency of oral administration [26], low bioavailability [27], and low efficacy of IDO inhibitors hinder their application [28,29].

Many strategies have been used to solve the problems of IDO inhibitors, mainly including structural optimization [30] and modification [31,32], and drug delivery, such as the IDO inhibitor-loaded nanoparticles [33], MOF [34], nanosuspensions [35], supramolecular prodrug nanocarriers [36], and micelles [37], among others. In our previous research, we constructed the sorafenib (SFN) nano-skeleton drug delivery system (MSNM@SFN), which could increase the solubility, dissolution, and oral bioavailability of SFN, showing enhanced anti-tumor effects compared with SFN suspension [38,39].

CY1-4, 9-nitropyridine [2′,3′:4,5] pyrimido [1,2-α] indole -5,11- dione, is a tryptanthrin derivative and a small molecule IDO inhibitor, which can effectively inhibit IDO-mediated immune resistance. However, CY1-4 is a poorly water-soluble substance, which seriously affects the absorption of CY1-4 along the gastrointestinal tract, resulting in low bioavailability and poor anti-tumor effect. Based on the previous research, we used the carrier material Sylysia with high dispersion function, HPMC with a crystal precipitation-inhibiting effect, and Distearoylphosphatidyl-ethanolamine-poly(ethylene glycol)_2000_ (DSPE-PEG_2000_) with a dissolution and absorption-promoting effect to prepare the CY1-4 nano-skeleton drug delivery system (MSNM@CY1-4) for improving the solubility, dissolution, and bioavailability of CY1-4, and investigating the anti-tumor activity and immune mechanism of MSNM@CY1-4.

## 2. Materials and Methods

### 2.1. Materials

CY1-4 was synthesized by our laboratory [40], and the detail for the synthesis of CY1-4 is shown in the Supporting Information. Mesoporous silica material (Sylysia^®^350, Sylysia) was purchased from Fuji Silysia Chemicals (Kasugai, Japan). Hydroxypropyl methylcellulose (HPMC) was obtained from Alfa Aesar Chemical Co., Ltd. (Tianjin, China). Distearoylphosphatidyl-ethanolamine-poly(ethylene glycol)_2000_ (DSPE-PEG_2000_) was provided by NOF (Shanghai, China) Co., Ltd. (Tokyo, Japan). L-Kynurenine and L-Tryptophan were supplied by MedChemExpress (Beijing, China). Dulbeco’s Modified Eagle Medium (DMEM), Trypsin-EDTA solution (0.25%), penicillin–streptomycin, and Fetal bovine serum (FBS) were all purchased from GIBCO, Invitrogen Corp. (Carlsbad, CA, USA). Fc blocking antibody (CD16/CD32 antibody), CD3e-PE antibody, CD4-FITC antibody, CD8a-APC-eFluor780 antibody, and Foxp3-PECy5 antibody were all obtained from eBioscience (San Diego, CA, USA). Collagenase IV, Hyaluronase, and DNAase were all provided by Sigma-Aldrich (St. Louis, MO, USA). All other chemicals were of analytical or high-performance liquid chromatography (HPLC) grade and were used without further purification.

The B16-F10 murine melanoma cell lines were purchased from the cell bank of the Chinese Academy of Sciences (Shanghai, China). The cells were cultivated in DMEM supplemented with 10% FBS and 1% penicillin–streptomycin and maintained at 37 °C under a humidified 5% CO_2_ atmosphere.

Female Sprague Dawley (SD) rats (190–210 g) and female C57BL/6 mice (18–20 g) were purchased from the Experimental Animal Center of Peking University Health Science Center. Female SD rats were used to study the in vivo pharmacokinetics of the preparations. The female C57 mice were used to study the IDO inhibition ability, in vivo anti-tumor effect and anti-tumor immune response of the preparations. Rats were housed in three per cage in the laboratory. The mice were housed in five per cage in the laboratory. All animals were housed in a specific pathogen-free environment with free access to standard food and water, maintained on a 12 h light/dark cycle under conditions of 25 ± 3 °C and 50 ± 5% humidity, and acclimatized for 7 days. At the end of the study, the animals were sacrificed with a cervical dislocation method following anesthesia with 1.5% isoflurane. All animal care and procedures were carried out in accordance with the guidelines approved by the Biomedical Ethics Committee of Peking University. The ethics approval numbers were DLASBD0402 and DLASBD0403.

### 2.2. Preparation of MSNM@CY1-4

MSNM@CY1-4 was prepared with a solvent evaporation method, as the ratio of CY1-4:Sylysia:HPMC:DSPE-PEG_2000_ (1:3:3:3 (*w/w/w/w*)). Briefly, CY1-4 was dissolved with the mixed solution of dichloromethane and anhydrous methanol (dichloromethane–anhydrous methanol = 3:1, *v/v*), Sylysia was dispersed with dichloromethane, HPMC was dispersed with dichloromethane, and DSPE-PEG_2000_ was dissolved with dichloromethane. After that, the CY1-4 solution was dropped into Sylysia dispersion liquid under magnetic stirring, and then sonicated for 30 min. Then, the HPMC dispersion liquid was dropped into the CY1-4-Sylysia mixture, stirred for 24 h, and evaporated to dryness under reduced pressure at 40 °C. Subsequently, the DSPE-PEG_2000_ solution was added, ultrasonicated for 15 min, and evaporated to dryness under reduced pressure at 40 °C to obtain MSNM@CY1-4.

### 2.3. Solubility of CY1-4 in MSNM@CY1-4

The excess MSNM@CY1-4 was placed in deionized water and was shaken in a 37 °C water bath for 48 h. The supernatant was filtered after centrifugation at 10,000 rpm, and the filtrate was collected. The CY1-4 was measured using HPLC to calculate the solubility of CY1-4.

### 2.4. In Vitro Dissolution of CY1-4 from MSNM@CY1-4

MSNM@CY1-4 (equivalent to 3 mg of CY1-4) was dispersed in 600 mL artificial gastric fluid (pH 1.2 containing 0.1% (*v/v*) Tween-80) or 600 mL artificial intestinal fluid (pH 6.8 containing 0.1% (*v/v*) Tween-80) at 37 °C and 100 rpm. Samples were taken at 0, 2, 5, 12, 20, 40, and 60 min. Each sample was filtered through a 0.22 μm filter membrane and determined by HPLC.

### 2.5. In Vivo Pharmacokinetics

Fifteen female SD rats (190–210 g) were fasted for at least 24 h prior to the experiment and were given water freely. The SD rats were randomly divided into three groups with five rats in each group. The first group was given a 50 mg/kg CY1-4 suspension. The second group was given MSNM@CY1-4 at a dose of 10 mg/kg CY1-4. The third group was given MSNM@CY1-4 at a dose of 20 mg/kg CY1-4. After intragastric administration, a volume of 0.5 mL blood was collected at 0.5, 1, 2, 4, 6, 8, 12, and 24 h, and then centrifuged at 10,000 rpm for 5 min. The plasma was taken and placed in the 1.5 mL EP tube, and then anhydrous methanol was added to precipitate protein. The sample was centrifuged at 10,000 rpm for 10 min, the supernatant was taken out, dried with nitrogen, and anhydrous methanol was added to ultrasonic redissolution, and the supernatant was analyzed by HPLC. The pharmacokinetic parameters were calculated by a non-compartment model of Kinetica 4.4.1 software.

### 2.6. In Vivo Anti-Tumor Efficacy

To study the in vivo anti-tumor effect, the B16F10 tumor-bearing C57 mice were established. The 24 female C57 mice (18–20 g) were subcutaneously injected in the armpit with 100 μL cell suspension containing 1 × 10^7^ B16F10 cells/mL. When the tumor volume approximated to 100 mm^3^, the B16F10 tumor-bearing mice were randomly subdivided into four groups (n = 6 per group). Each experimental group was given the following four preparations by daily oral administration for 9 days: The first group was given saline. The second group was given 50 mg/kg CY1-4 suspension. The third group was given MSNM@CY1-4 at a dose of 10 mg/kg CY1-4. The fourth group was given MSNM@CY1-4 at a dose of 20 mg/kg CY1-4. The length and width of tumor in each group of mice were measured with a vernier caliper every two days. The relative tumor volume was calculated according to the formula, V = length × width^2^ × 0.5 (mm^3^), and the graph of relative tumor volume–time was drawn. The weight of tumor-bearing mice was weighed with the electronic balance every two days. The weight–time diagram of tumor-bearing mice was drawn. The survival time of tumor-bearing mice was recorded.

### 2.7. IDO Inhibition Assays

The ratio of kynurenine to tryptophan was measured by HPLC to explore the activity of IDO enzyme in the plasma and tumor of B16F10 tumor-bearing mice. The female C57 mice were subcutaneously injected in the armpit with 100 μL cell suspension containing 1 × 10^7^ B16F10 cells/mL. When the tumor volume approximated to 100 mm^3^, the B16F10 tumor-bearing mice were randomly subdivided into three groups (n = 3 per group). Each experimental group was given the following three preparations by daily oral administration for 6 days: The first group was given saline. The second group was given 50 mg/kg CY1-4 suspension. The third group was given MSNM@CY1-4 at a dose of 20 mg/kg CY1-4.

On day 14 after tumor inoculation, the blood samples were taken out and 100 μL serum samples were obtained by centrifugation at 5000 rpm for 5 min. Afterwards, 60 μL of 6% perchloric acid was added to the serum, covered with a lid, and mixed evenly on the vortex mixer. The mixtures were placed at room temperature for 15 min to fully precipitate the protein in the serum. The mixtures were centrifuged at 10,000× *g* for 10 min, and then the supernatants were collected for HPLC detection.

On day 14 after tumor inoculation, all the mice were sacrificed, the tumor tissues were harvested, photographed, and weighed, and then homogenized with homogenizer. Afterwards, the mixed solution of water/trichloroacetic acid/acetonitrile (62%/30%/8%) was added to the tumor homogenate and mixed evenly on the vortex mixer. The mixtures were treated at 50 °C for 30 min to completely hydrolyze N-formyl kynurenine. The mixtures were centrifuged at 10,000× *g* for 10 min, and then the supernatants were collected and transferred to another Eppendorf tube. Under the same conditions, the supernatants were further centrifuged for 10 min to make the precipitations more complete, and the new supernatants were collected for HPLC detection.

The HPLC detection was performed on a Luna C18 column (250 mm × 4.6 mm, 5 μm, Phenomenex, 30 °C) with acetonitrile–water = 10:90 (aqueous phase of 15 mM, pH = 4.0 sodium acetate solution) as the mobile phase at a flow rate of 1 mL/min. The detection wavelength of Kyn and Trp was set to 360 nm and 280 nm, respectively.

### 2.8. In Vivo Anti-Tumor Immune Flow Cytometric Analysis

The female C57 mice were subcutaneously injected in the armpit with 100 μL cell suspension containing 1 × 10^7^ B16F10 cells/mL. When the tumor volume approximated to 100 mm^3^, the B16F10 tumor-bearing mice were randomly subdivided into three groups (n = 3 per group). Each experimental group was given the following three preparations by daily oral administration for 9 days: The first group was given saline (control). The second group was given 50 mg/kg CY1-4 suspension. The third group was given MSNM@CY1-4 at a dose of 20 mg/kg CY1-4. On day 17 after tumor inoculation, the inguinal lymph nodes and tumor tissues of mice in the control group, CY1-4 suspension group, and MSNM@CY1-4 group were taken out, and the single-cell suspensions of tumor drainage lymph nodes and tumor tissues in each group were prepared.

The single-cell suspensions of the above two tissues were incubated with Fc blocking antibody. CD3e^+^CD4^+^ T cells (stained with CD3e-PE antibody and CD4-FITC antibody), CD3e^+^CD8a^+^ T cells (stained with CD3e-PE antibody and CD8a-APC-eFluor780 antibody), and Tregs (stained with CD4-FITC antibody and Foxp3-PECy5 antibody) in inguinal lymph nodes and tumor tissues were analyzed via Gallios Flow cytometric analysis.

### 2.9. HPLC Analysis of CY1-4

The concentration of CY1-4 was determined by HPLC. A Shimadzu liquid chromatograph LC-20A was used for measurement. A Luna C18 column (250 mm × 4.6 mm, 5 μm, Phenomenex) was used and the wavelength was set at 258 nm. The mobile phase consisted of acetonitrile–deionized water (30:70, *v/v*), and the flow rate was 1 mL/min. The column temperature was 40 °C.

### 2.10. Statistical Analysis

SPSS 22.0 statistical software was used to process the data. All data are reported as the means ± standard deviations from at least three repeated experiments. One-way analysis of variance (ANOVA) was used to determine the significance between groups, and then a Bonferroni corrected post hoc test was used for comparison between groups. The value of *p* < 0.05 or less indicated significance.

## 3. Results

### 3.1. Solubility of CY1-4 in MSNM@CY1-4

**Figure 1:**
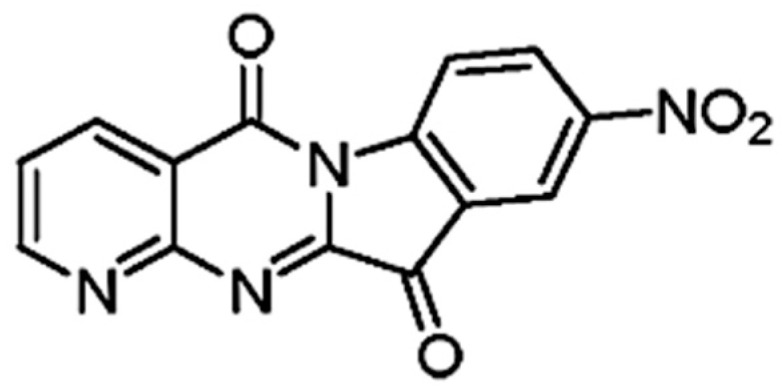
The chemical structural formula of CY1-4.

The chemical structural formula of CY1-4, 9-nitropyridine [2′,3′:4,5] pyrimido [1,2-α] indole-5,11-dione, is shown in Figure 1, and its molecular weight is 294 g/mol.

According to the determination in Table 1, the solubility of CY1-4 in deionized water was 1.16 μg/mL, less than 0.1 mg/mL, which made it an insoluble drug. However, the solubility of CY1-4 in MSNM@CY1-4 was 233.22 μg/mL, nearly 200 times that of CY1-4, showing that MSNM@CY1-4 could significantly improve the solubility of CY1-4.

The influence of Sylysia, HPMC, and DSPE-PEG_2000_ on CY1-4 solubility was also investigated. As shown in Supplementary Table S2, the solubility of CY1-4 in a CY1-4-Sylysia nano-skeleton was 25.47 μg/mL, which was 22.0 times that of CY1-4. The solubility of CY1-4 in the CY1-4-Sylysia-HPMC nano-skeleton increased to 36–74 μg/mL, which was 33–63 times that of CY1-4. With the increase in HPMC content, the solubility of CY1-4 also increased. The solubility of CY1-4 in MSNM@CY1-4 was significantly higher than that of CY1-4 and other prescriptions. The compatibilization effect of MSNM@CY1-4 was the best, which was the optimal prescription. The subsequent in vitro and in vivo studies will be conducted on MSNM@CY1-4. In addition, the characterizations of MSNM@CY1-4 were shown in the supporting information and Supplementary Figures S2–S4 and Table S3.

### 3.2. In Vitro Dissolution of CY1-4 from MSNM@CY1-4

The dissolution and absorption process of the oral drug delivery system is mainly carried out in the gastrointestinal tract, and different parts of the gastrointestinal tract have different pH values. We selected two typical gastrointestinal tract parts, namely gastric juice and small intestinal juice, and simulated the pH environment of gastric juice and small intestinal juice by pH 1.2 and pH 6.8 buffers to investigate the dissolution rate of MSNM@CY1-4.

**Figure 2:**
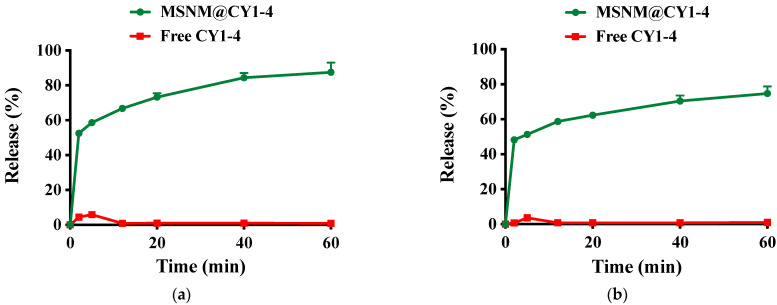
In vitro release of CY1-4 from MSNM@CY1-4 in (**a**) artificial gastric fluid (pH 1.2) and (**b**) artificial intestinal fluid (pH 6.8).

The dissolution of CY1-4 from MSNM@CY1-4 is shown in Figure 2. As shown in Figure 2a, CY1-4 was rapidly dissolved from MSNM@CY1-4 in the artificial gastric fluid of pH 1.2. About 60% of CY1-4 was dissolved from MSNM@CY1-4 at 5 min, and about 88% of CY1-4 was dissolved from MSNM@CY1-4 at 60 min. However, the dissolution rate of CY1-4 in Free CY1-4 was much lower than that of CY1-4 in MSNM@CY1-4. The maximum dissolution of CY1-4 in Free CY1-4 was about 6% at 5 min and then decreased to about 1%. As shown in Figure 2b, CY1-4 was rapidly dissolved from MSNM@CY1-4 in the artificial intestinal fluid of pH 6.8. About 51% of CY1-4 was dissolved from MSNM@CY1-4 at 5 min, and about 75% of CY1-4 was dissolved from MSNM@CY1-4 at 60 min. However, the dissolution rate of CY1-4 in Free CY1-4 was much lower than that of CY1-4 in MSNM@CY1-4. The maximum dissolution of CY1-4 in Free CY1-4 was about 4% at 5 min and then decreased to about 1%.

The dissolution rate of the drug is one of the key factors affecting the absorption of the oral drug delivery system. According to the Noyes–Whitney equation, reducing the particle size, increasing the surface area, and improving the solubility of the drug can increase the dissolution rate of the drug. Compared with CY1-4, the high dispersion of Sylysia in MSNM@CY1-4 and the large number of nano-scale pores cause CY1-4 to have a larger surface area and smaller particle size, and the solubility of MSNM@CY1-4 is much higher than that of CY1-4. Therefore, compared with free CY1-4, the dissolution rate of CY1-4 in MSNM@CY1-4 in artificial gastric juice and artificial intestinal juice was faster and more sufficient, which was beneficial to the absorption of CY1-4 in the gastrointestinal tract.

### 3.3. In Vivo Pharmacokinetics

**Figure 3:**
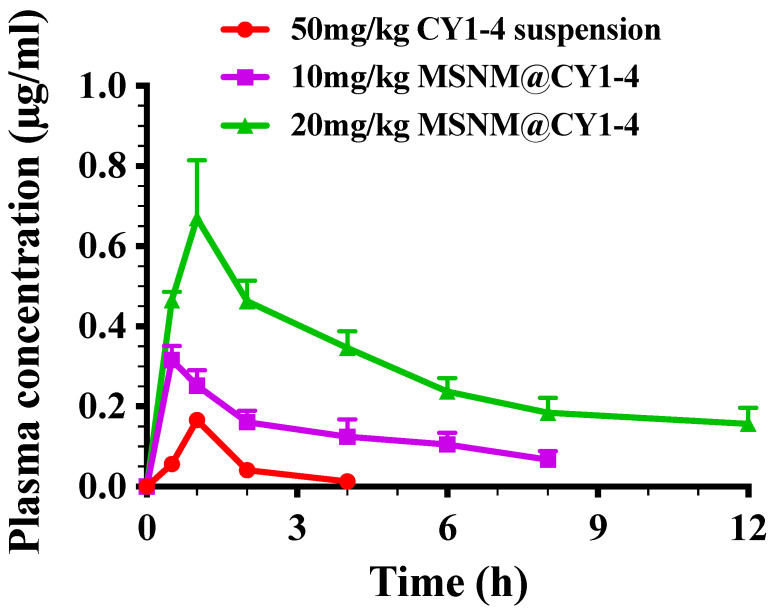
In vivo plasma concentration–time curve of CY1-4 after oral administration of MSNM@CY1-4.

The pharmacokinetic profile of MSNM@CY1-4 was examined in Sprague Dawley (SD) rats. The doses of MSNM@CY1-4 were 10 mg/kg and 20 mg/kg, respectively, and the dose of CY1-4 suspension was 50 mg/kg. After a single oral administration of MSNM@CY1-4, the plasma concentration of CY1-4 was measured by HPLC, and the average plasma concentration–time curve is plotted (Figure 3). After oral administration of 20 mg/kg MSNM@CY1-4, CY1-4 was rapidly absorbed, and the maximum plasma drug concentration was observed 1 h post-dose and remained detectable within 12 h. The plasma concentration of CY1-4 in the 20 mg/kg MSNM@CY1-4 group was significantly higher than that in the 10 mg/kg MSNM@CY1-4 and 50 mg/kg CY1-4 suspension groups, and the plasma concentration of CY1-4 in the 10 mg/kg MSNM@CY1-4 group was significantly higher than that in the 50 mg/kg CY1-4 suspension group.

The pharmacokinetic parameters of MSNM@CY1-4 after a single oral administration in rats are shown in Table 2. The C_max_ of CY1-4 in the 20 mg/kg MSNM@CY1-4 group was about 2.1-fold more than that in 10 mg/kg MSNM@CY1-4 group (*p* < 0.01), and about 3.9-fold more than that in 50 mg/kg CY1-4 suspension group (*p* < 0.01). The C_max_ of CY1-4 in the 10 mg/kg MSNM@CY1-4 group was about 1.9-fold more than that in the 50 mg/kg CY1-4 suspension group (*p* < 0.05). There was no significant difference in T_max_ of CY1-4 among the 20 mg/kg MSNM@CY1-4, 10 mg/kg MSNM@CY1-4, and 50 mg/kg CY1-4 suspension groups. The AUC_0–∞_ of CY1-4 in the 20 mg/kg MSNM@CY1-4 group was about 3.7-fold more than that in the 10 mg/kg MSNM@CY1-4 group (*p* < 0.01), and about 23.9-fold more than that in the 50 mg/kg CY1-4 suspension group (*p* < 0.01). The AUC_0–∞_ of CY1-4 in the 10 mg/kg MSNM@CY1-4 group was about 6.5-fold more than that in the 50 mg/kg CY1-4 suspension group (*p* < 0.05). The T_1/2_ of CY1-4 in 20 mg/kg MSNM@CY1-4 group was about 3.5-fold more than that in the 10 mg/kg MSNM@CY1-4 group (*p* < 0.01), and about 11.4-fold more than that in the 50 mg/kg CY1-4 suspension group (*p* < 0.01). The T_1/2_ of CY1-4 in the 10 mg/kg MSNM@CY1-4 group was about 3.2-fold more than that in the 50 mg/kg CY1-4 suspension group (*p* < 0.05).

The pharmacokinetic profiles of MSNM@CY1-4 suggested that the formation of the nano-skeleton delivery system increases the plasma concentration of CY1-4 and elongates the blood circulation compared with CY1-4 suspension. Compared with CY1-4 suspension, the bioavailability of CY1-4 in MSNM@CY1-4 was significantly improved, and the bioavailability of 20 mg/kg MSNM@CY1-4 was about 23.9-fold more than that in the 50 mg/kg CY1-4 suspension.

### 3.4. In Vivo Anti-Tumor Efficacy

**Figure 4:**
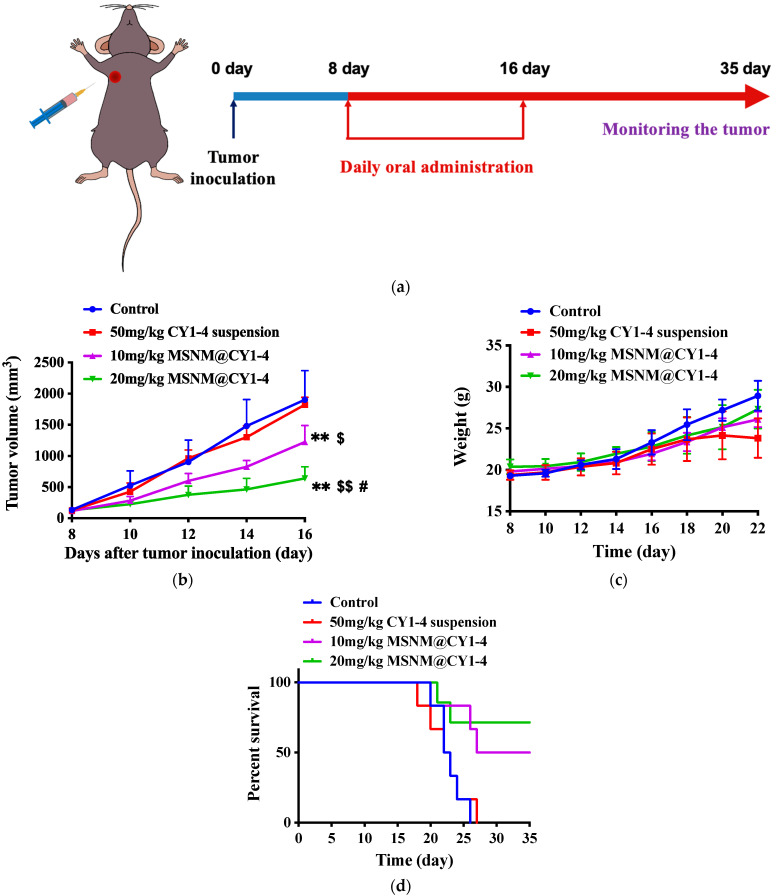
In vivo anti-tumor study of MSNM@CY1-4 in B16F10 tumor-bearing C57BL/6 mice. (**a**) Schematic graph of the experimental design. (**b**) Tumor volume curves of tumor-bearing mice received different treatments. (**c**) Body weight changes of the tumor-bearing mice. (**d**) Survival curve of tumor-bearing mice received different treatments. Data are shown as mean ± SD, n = 6 (** *p* < 0.01 vs. control; $ *p* < 0.05, $$ *p* < 0.01 vs. 50 mg/kg CY1-4 suspension; # *p* < 0.05 vs. 10 mg/kg MSNM@CY1-4).

The anti-tumor effect of MSNM@CY1-4 was evaluated in a B16F10 tumor-bearing C57BL/6 mouse model. On the eighth day after tumor inoculation, the tumor volume reached 100 mm^3^, and the tumor-bearing mice were randomly divided into four groups (n = 6). Mice were orally administered with saline, 50 mg/kg CY1-4 suspension, 10 mg/kg MSNM@CY1-4, and 20 mg/kg MSNM@CY1-4 once a day for 9 days (Figure 4a). The tumor volume, body weight, and survival time of mice were measured during the experiment.

The tumor volume–time curve of B16F10 tumor-bearing C57BL/6 mice in each administration group is shown in Figure 4b. The 20 mg/kg MSNM@CY1-4 group generated optimal inhibition of tumor growth compared with the control, 50 mg/kg CY1-4 suspension, and 10 mg/kg MSNM@CY1-4 groups (*p*  <  0.01 or *p*  <  0.05). The 10 mg/kg MSNM@CY1-4 group showed moderate inhibition capability compared with the control and 50 mg/kg CY1-4 suspension groups (*p*  <  0.01 or *p*  <  0.05). The 50 mg/kg CY1-4 suspension group did not elicit significant tumor inhibition ability compared with the control group. On the 16th day after tumor inoculation, the average tumor sizes of the control, 50 mg/kg CY1-4 suspension, 10 mg/kg and 20 mg/kg MSNM@CY1-4 groups were 1901 ± 470 mm^3^, 1822 ± 119 mm^3^, 1226 ± 262 mm^3^, and 640 ± 187 mm^3^, respectively. The corresponding tumor growth inhibition rates in the 10 mg/kg and 20 mg/kg MSNM@CY1-4 groups were 35.5% and 66.3% compared with the control group, respectively. These results clarified that the 20 mg/kg MSNM@CY1-4 group elicited the best tumor-inhibiting efficacy.

The body weight–time curve of B16F10 tumor-bearing C57BL/6 mice in each administration group is shown in Figure 4c. The results showed that the weight of tumor-bearing mice in each administration group was an upward trend, and there was no serious weight loss, indicating the safety of MSNM@CY1-4 and CY1-4 suspension.

The survival curve of B16F10 tumor-bearing C57BL/6 mice in each administration group is shown in Figure 4d. The mice in the control group began to die on the 20th day after tumor inoculation, and all died on the 26th day after tumor inoculation, with a mortality rate of 100%. The mice in the 50 mg/kg CY1-4 suspension group began to die on the 18th day after tumor inoculation, and all died on the 27th day after tumor inoculation, with a mortality rate of 100%. The mice in the 10 mg/kg MSNM@CY1-4 group began to die on the 20th day after tumor inoculation, and the mortality rate was 50% on the 38th day after tumor inoculation. The mice in the 20 mg/kg MSNM@CY1-4 group began to die on the 23rd day after tumor inoculation, and the mortality rate was 17% on the 38th day after tumor inoculation, which was significantly lower than that of other groups. The results indicated that the 20 mg/kg MSNM@CY1-4 group significantly prolonged the survival time of B16F10 tumor-bearing mice compared with the control, 50 mg/kg CY1-4 suspension and 10 mg/kg MSNM@CY1-4 groups, and 20 mg/kg MSNM@CY1-4 group had the best therapeutic effect, which was consistent with the results obtained from tumor growth inhibition.

The 20 mg/kg MSNM@CY1-4 group could significantly improve the solubility, dissolution, and bioavailability of CY1-4 and had the best anti-tumor effect. MSNM@CY1-4 increased the anti-tumor activity of CY1-4, and the superior anti-tumor performance of 20 mg/kg MSNM@CY1-4 can be attributed to the improvement of bioavailability of CY1-4 on the one hand, and on the other hand, it may be related to CY1-4-mediated IDO inhibition and related immune responses. The subsequent anti-tumor investigation will be conducted on the 20 mg/kg MSNM@CY1-4, 50 mg/kg CY1-4 suspension, and control groups.

### 3.5. IDO Inhibition Assays

**Figure 5:**
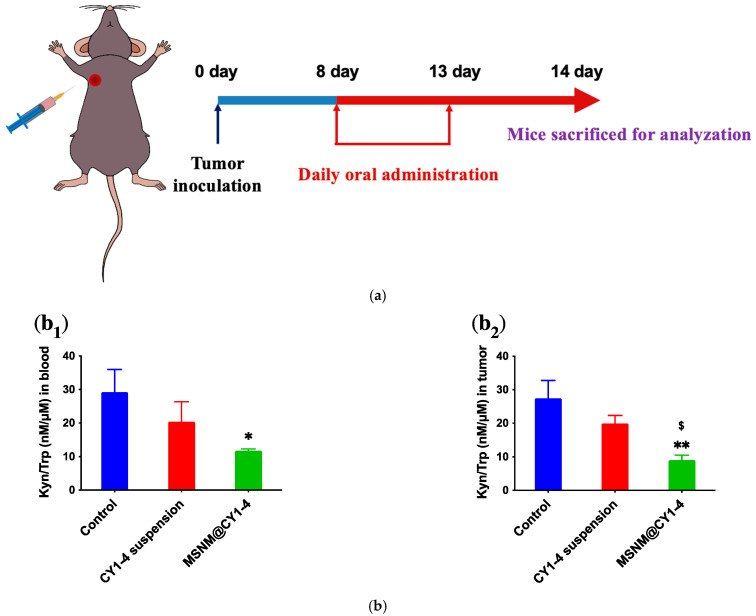

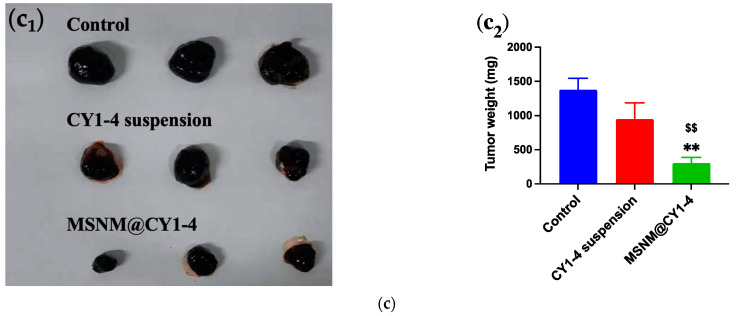
In vivo IDO inhibition study of MSNM@CY1-4 in B16F10 tumor-bearing C57BL/6 mice. (**a**) Schematic graph of the experimental design. (**b**) The Kyn/Trp ratio in (**b_1_**) blood and (**b_2_**) tumor following various treatments. (**c**) The photograph images (**c_1_**) and weight (**c_2_**) of tumors collected from mice on the 14th day after tumor inoculation. Data are shown as mean ± SD, n = 3 (* *p* < 0.05, ** *p* < 0.01 vs. control; $ *p* < 0.05, $$ *p* < 0.01 vs. CY1-4 suspension).

IDO is a typical metabolic enzyme and overexpressed in many malignant tumors, which can catalyze the decomposition of tryptophan (Trp) into kynurenine (Kyn) and mediate tumor immunosuppression [41]. CY1-4 is a small molecule IDO inhibitor, which can inhibit IDO and alleviate immunosuppression. MSNM@CY1-4 was absorbed into the blood circulation from the gastrointestinal tract and reached the tumor site. In order to detect the IDO inhibitory effect of MSNM@CY1-4 in blood and tumor, the B16F10 tumor-bearing animal model was established on C57BL/6 mice. On the eighth day after tumor inoculation, the tumor volume reached 100 mm^3^, and the tumor-bearing mice were randomly divided into three groups (n = 3). Mice were orally administered with saline, 50 mg/kg CY1-4 suspension, and 20 mg/kg MSNM@CY1-4 once a day for 6 days (Figure 5a). On the 14th day after tumor inoculation, Trp and Kyn in blood and tumor tissues were measured by HPLC, the ratio of Kyn to Trp was calculated, and the inhibitory effect of MSNM@CY1-4 on IDO was evaluated.

The ratio of Kyn/Trp in blood is shown in Figure 5b_1_. The results showed that the ratio of Kyn/Trp in the MSNM@CY1-4 group was about 0.4-fold more than that in the control group (*p* < 0.01), and the ratio of Kyn/Trp in the MSNM@CY1-4 group was lower than that in the 50 mg/kg CY1-4 suspension group, indicating that MSNM@CY1-4 could effectively block the metabolism of Trp to Kyn and effectively inhibit IDO activity. In addition, the ratio of Kyn/Trp in the CY1-4 suspension group was lower than that in the control group, and there was no significant difference, indicating that CY1-4 suspension could reduce the metabolism of Trp to Kyn, but the inhibitory effect was low.

The ratio of Kyn/Trp in tumor tissues is shown in Figure 5b_2_. The results showed that the ratio of Kyn/Trp in the MSNM@CY1-4 group was about 0.3-fold more than that in the control group (*p* < 0.01), and the ratio of Kyn/Trp in the MSNM@CY1-4 group was about 0.5-fold more than that in the CY1-4 suspension group (*p* < 0.05), indicating that MSNM@CY1-4 could effectively block the metabolism of Trp to Kyn and effectively inhibit the activity of IDO in the tumor site, which was contributed to reduce IDO-mediated immune resistance and overcome the immunosuppressive tumor microenvironment. In addition, the ratio of Kyn/Trp in the CY1-4 suspension group was lower than that in the control group, and there was no significant difference, indicating that CY1-4 suspension could reduce the metabolism of Trp to Kyn, but the inhibitory effect was low.

The tumor anatomical entity image and tumor weight of B16F10 tumor-bearing mice after oral administration of MSNM@CY1-4 are also shown in Figure 5c. The results showed that on the 14th day after tumor inoculation, the tumors in the MSNM@CY1-4 group were significantly reduced, the average tumor weight in the MSNM@CY1-4 group was significantly lower than that in the control and CY1-4 suspension groups (*p* < 0.01), indicating that MSNM@CY1-4 could significantly inhibit tumor growth and elicited the best anti-tumor effect, which was consistent with the previous anti-tumor efficacy results.

### 3.6. In Vivo Anti-Tumor Immune Flow Cytometric Analysis

**Figure 6:**
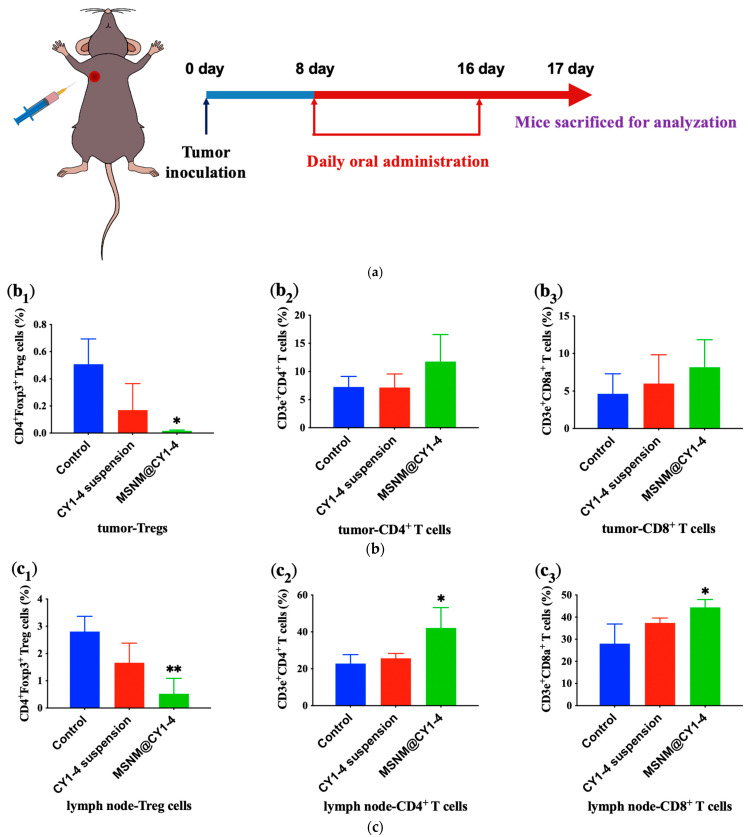
In vivo anti-tumor immunity response of MSNM@CY1-4 in B16F10 tumor-bearing C57BL/6 mice. (**a**) Schematic illustration of the experimental design. (**b**) Infiltration of (**b_1_**) Tregs, (**b_2_**) CD4^+^ T cells, and (**b_3_**) CD8^+^ T cells in tumors through the flow cytometric examination. (**c**) Tumor-draining lymph node infiltration of (**c_1_**) Tregs, (**c_2_**) CD4^+^ T cells, and (**c_3_**) CD8^+^ T cells through flow cytometric examination. Data are shown as mean ± SD, n = 3 (* *p* < 0.05, ** *p* < 0.01 vs. control).

IDO is highly expressed in the tumor microenvironment (TME), the introduction of CY1-4 into the nano-skeleton delivery system dramatically increased the IDO inhibition ability, and greatly reduced the Kyn/Trp ratio. Next, we further evaluated the anti-tumor immune response induced by IDO blockade in B16F10 tumor-bearing C57BL/6 mice. On the eighth day after tumor inoculation, the tumor volume reached 100 mm^3^, and the tumor-bearing mice were randomly divided into three groups (n = 3). Mice were orally administered with saline, 50 mg/kg CY1-4 suspension, and 20 mg/kg MSNM@CY1-4 once a day for 9 days (Figure 6a). On the 17th day after tumor inoculation, the immune cells in the tumor and tumor-draining lymph nodes of mice were measured by flow cytometry.

The intratumoral infiltration of regulatory T cells (Tregs) has been correlated with the poor prognosis of cancer patients [42,43]. We thus examined the intratumor localization of Tregs in tumor-bearing mice after MSNM@CY1-4 administration. The high intratumoral infiltration of Tregs was detected in the control group, implying B16F10 tumors have an immunosuppressive microenvironment (Figure 6b). After administration of MSNM@CY1-4, the proportion of Tregs infiltrated in tumors decreased significantly (*p* < 0.05), and the proportion of CD4^+^ T cells and CD8^+^ T cells increased, which was higher than that of the CY1-4 suspension group, indicating that MSNM@CY1-4 effectively suppressed IDO-mediated immune resistance and enhanced anti-tumor immune response, thereby inhibiting the rapid progression of tumors [41,44,45,46].

In addition to tumor microenvironment, IDO inhibitors also play an important role in tumor-draining lymph nodes (TDLNs) [47]. Because IDO is overexpressed not only in tumors, but also in antigen presenting cells (APCs) [48,49]. APCs are primarily localized at the lymphatic systems, including lymphatic nodes. The high expression of IDO will accelerate the metabolism of tryptophan to kynurenine, induce anergy of effector T cells, and shift CD4^+^ T cells to Treg cells during T-cell priming [50,51]. Therefore, we further evaluated the effect of MSNM@CY1-4 on immune cells infiltrated by TDLN (Figure 6c).

The infiltration of Tregs in TDLN in the control group was high, which was consistent with the results reported in the literature [52,53]. After the administration of MSNM@CY1-4, the proportion of Tregs infiltrated in tumor was significantly decreased (*p* < 0.01), and the proportion of CD4^+^ T cells and CD8^+^ T cells was significantly increased (*p* < 0.05), which was higher than that of the CY1-4 suspension group, indicating that MSNM@CY1-4 can reach the TDLN and block IDO activity during T-cell priming.

In addition, compared with the control group, there was no significant difference in the proportion of infiltrating Tregs, CD4^+^ T cells and CD8^+^ T cells in tumor tissues, and TDLN of mice in CY1-4 suspension group, which was attributed to the weak IDO inhibition of CY1-4 suspension. The poor activity of CY1-4 suspension to inhibit IDO and the inability to effectively initiate an anti-tumor immune response may be the reason that CY1-4 suspension failed to inhibit tumor growth.

## 4. Discussion

CY1-4 also has the problem of poor water solubility, which is the same as the IDO inhibitor that entered clinical research [54]. CY1-4 existed in irregular crystalline form (Figure S2a,b), and its solubility in water was poor. We used Sylysia as the carrier of CY1-4. As a common mesoporous silica nano-skeleton material, Sylysia has a large specific surface area and nano-scale pores of about 21 nm (Figure S4 and Table S3), which can highly disperse CY1-4 and limit the growth of crystals in the pores, so that poorly water-soluble CY1-4 exists in an amorphous state that is easy to dissolve [55]. However, the solubilization effect of CY1-4-Sylysia nano-skeleton was limited, which may be due to the fact that CY1-4 was easy to transform into a crystalline state during the dissolution process [56,57]. We used hydrophilic polymer HPMC and lipid DSPE-PEG_2000_ as coating materials. HPMC is the most widely used biodegradable hydrophilic polymer, which can increase the wettability of poorly water-soluble drugs and inhibit the formation and growth of CY1-4 crystals during the dissolution process and storage [58,59,60]. Amphiphilic polymer DSPE-PEG_2000_ can inhibit the precipitation of CY1-4 during the dilution process [61,62]. The crystallization and crystallization peaks of CY1-4 were not found in MSNM@CY1-4 (Figures S2d and S3), and CY1-4 existed in an amorphous state, which may be the reason for the increase in solubility of CY1-4.

For oral administration, the effect of the drug depends largely on its oral bioavailability [63,64]. The significant increase in bioavailability of MSNM@CY1-4 was attributed to the significant increase in solubility and dissolution on the one hand, and on the other hand, it may be related to the inhibition of intestinal transporters by DSPE-PEG_2000_ and the promotion of the absorption of poorly water-soluble drugs in the gastrointestinal tract [65,66].

IDO inhibitors entering clinical research have the problems of large dosage and high frequency of oral administration [67]. For example, in the phase II trial (NCT02077881) of indoximod (IND), the dosage of oral administration reached 1200 mg twice a day [68]. In the phase I clinical trial of IND (NCT00567931), the dosage of oral administration reached 2000 mg twice a day [69]. PF-06840003 up to 500 mg BID was generally well tolerated with evidence of a pharmacodynamic effect and durable clinical benefit in a subset of patients with recurrent malignant glioma [70]. Epacadostat was well tolerated and could effectively inhibit the activity of IDO1 at a dose of ≥100 mg BID [71]. In order to solve the problem, Wu et al. prepared oral indoximod (IND) nanoparticles (IND-NPs), and the dosage of 200 mg/kg of IND nanoparticles could effectively inhibit ulcerative colitis in mice [72]. Calleja P et al. prepared Indoximod (1-MT) nanocrystals, and the tumor growth inhibition rate of oral administration of 100 mg/kg of 1-MT nanocrystals was about 40% in the Lewis lung cancer mouse model [73]. Our results showed that MSNM@CY1-4 with a CY1-4 dosage of 10 mg/kg and 20 mg/kg showed a good anti-tumor effect, and its tumor growth inhibition rates were 35.5% and 66.3% respectively.

In 2018, the phase III clinical trial of epacadostat (IDO inhibitor, Incyte) combined with Keytruda (PD-1 inhibitor, Merck) in the treatment of unresectable metastatic melanoma failed. Compared with Keytruda alone, the combination immunotherapy failed to significantly improve the progression-free survival. On the one hand, the failure of the experiment may be due to the inapplicability of pharmacodynamic indicators or the mismatch of drug combination strategies. On the other hand, it may be that the exact regulatory mechanism of IDO1 in physiology and pathology and its impact on the tumor microenvironment are not well understood [74], and tumor cells or immune cells in the tumor microenvironment may express other tryptophan-degrading enzymes beyond IDOs (i.e., TDO), thus still escaping from immune surveillance in the case of IDO inhibition [75,76]. TDO is still necessary to determine whether IDO selective inhibitors are sufficient, as TDO, which catalyzes the same reaction as IDOs, is up-regulated in a variety of tumors, such as breast cancer, cervical cancer, glioblastoma, colorectal cancer, bladder cancer, lung cancer, and hepatocellular carcinoma, promoting immunosuppression and improving tumor invasiveness [77,78]. Epacadostat selectively blocks the expression of IDO1, but the TDO pathway can play a potential compensatory role in causing tumor immunosuppression [79]. In order to solve this problem, IDO/TDO dual inhibitors are currently being developed, such as LPM-3480226, DN-1406131, and SHR-9146, which have entered the clinical trials, but the specific structures have not been disclosed [80].

CY1-4 is a small molecule IDO/TDO inhibitor [40], which can effectively inhibit IDO/TDO-mediated immune resistance, relieve immunosuppressive TME and TDLN, significantly reduce Tregs, increase CD4^+^ T cells and CD8^+^ T cells, and improve the anti-tumor effect. Therefore, IDO/TDO-blocking immunotherapy mediated by CY1-4 may be a promising strategy to reduce tumor immune escape, overcome the deficiency of IDO inhibitors in clinical trials, and expand the application scope of cancer treatment.

## 5. Conclusions

In this present study, we constructed a CY1-4 nano-skeleton drug delivery system (MSNM@CY1-4) to improve the anti-tumor immune effect of CY1-4. Our results indicated that MSNM@CY1-4 can significantly increase the solubility, dissolution, and bioavailability of CY1-4. In the B16F10 tumor-bearing mouse model, MSNM@CY1-4 can significantly inhibit the activity of IDO in blood and tumor tissues, ameliorate the immunosuppressive TME and TDLN, significantly reduce Tregs infiltration, increase CD4^+^ T cells and CD8^+^ T cells infiltration, and improve anti-tumor effects. Our study provides a reference for increasing the solubility of oral poorly water-soluble drugs, optimizing pharmacokinetic properties, improving the efficacy and immune response, and providing benefits for cancer treatment.

## Figures and Tables

**Table 1:** The solubility of CY1-4.

	Solubility (μg/mL)
CY1-4	1.16 ± 0.08
MSNM@CY1-4 (CY1-4:Sylysia:HPMC:DSPE-PEG_2000_ = 1:3:3:3)	233.22 ± 8.94

**Table 2:** Pharmacokinetic parameters of CY1-4 after oral administration of MSNM@CY1-4 or CY1-4 suspension.

Parameters	CY1-4 Suspension 50 mg/kg	MSNM@CY1-4 10 mg/kg	MSNM@CY1-4 20 mg/kg
C_max_ (mg/L)	0.17 ± 0.01	0.32 ± 0.03 *	0.67 ± 0.14 ** **^##^**
T_max_ (h)	1.00 ± 0.00	0.60 ± 0.22	1.00 ± 0.00
AUC_(0–∞)_ (mg/L·h)	0.20 ± 0.03	1.29 ± 0.17 **	4.77 ± 0.89 ** **^##^**
T_1/2_ (h)	1.13 ± 0.06	3.64 ± 0.68 **	12.86 ± 1.29 ** **^##^**

Data are shown as mean ± SD, n = 5 (* *p* < 0.05, ** *p* < 0.01 vs. 50 mg/kg CY1-4 suspension; ## *p* < 0.01 vs. 10 mg/kg MSNM@CY1-4).

## Data Availability

The original contributions presented in the study are included in the article/Supplementary Material, further inquiries can be directed to the corresponding authors.

## Supplementary Materials

The following supporting information can be downloaded at: https://www.mdpi.com/article/10.3390/jfb15120372/s1, Figure S1: UV-vis spectra of CY1-4; Figure S2: Topical SEM images of (**a**) pure CY1-4 and (**b**) local amplification of pure CY1-4, (**c**) Sylysia and (**d**) MSNM@CY1-4; Figure S3: PXRD patterns of pure CY1-4, Sylysia, HPMC, DSPE-PEG_2000_, Physical mixtures and MSNM@CY1-4; Figure S4: The BJH pore size distribution curves of Sylysia and MSNM@CY1-4; Table S1: The solubility of CY1-4; Table S2: The solubility of MSNM@CY1-4; Table S3: Specific surface areas and pore volumes of Sylysia and MSNM@CY1-4.
