# Supplementary material for: Improving the Anti-Tumor Effect of Indoleamine 2,3-Dioxygenase Inhibitor CY1-4 by CY1-4 Nano-Skeleton Drug Delivery System
Source: J Funct Biomater. 2024 Dec 9;15(12):372. doi: 10.3390/jfb15120372 (PMC11676189; doi:10.3390/jfb15120372)
Supplement: Supplementary file 1 [file jfb-15-00372-s001.zip › jfb-3338124-supplementary.pdf]

Journal of
Functional Biomaterials

MDPI

Article

Improving the anti-tumor effect of indoleamine 2,3-dioxygenase inhibitor CY1-4 by CY1-4 nano-skeleton drug delivery system

Hui Li, Junwei Liu, Jingru Wang, Zhuoyue Li, Jianming Yu, Xu Huang, Bingchuan Wan, Xiangbao Meng * and Xuan Zhang *

1. Supplementary Methods

1.1. Synthesis of CY1-4

5-nitroisatin (0.9 mmol) and 1,8-diazabicyclo[5.4.0]undec-7-ene (DBU) (2 mmol) were dissolved in N,N-Dimethylformamide (DMF) (3 mL) and stirred at room temperature for 10 min as the reaction solution A. 2-aminonicotinic acid (1 mmol), N-Methylmorphine (1.8 mmol) and O-benzotriazole-N,N,N',N'-tetramethyl-uronium-hexafluorophosphate (HBTU) (1 mmol) were dissolved in DMF (6 mL) and stirred at room temperature for 10 min as the reaction solution B. The reaction solution A was slowly added into the reaction solution B, and stirred at room temperature for 20 h. The reaction was monitored by thin layer chromatography to determine that the reaction was complete. After the reaction was completed, the solvent was evaporated. The CY1-4 was obtained by column chromatography separation and purification (dichloromethane:methanol = 80:1, *v* / *v*), and the yield was about 42.4%.

1.2. Maximum absorption wavelength of CY1-4

The maximum absorption wavelength of CY1-4 was determined by UV/VIS spectrophotometer (UH5300, Hitachi, Japan). CY1-4 was accurately weighed and dissolved in acetonitrile to a standard solution with a concentration of 40 μg / mL. The absorbance of CY1-4 was measured in the 190-600 nm spectral region with acetonitrile as the reference solution, and the A-λ light absorption curve was drawn to determine the maximum absorption wavelength of CY1-4.

1.3. Solubility of CY1-4

The excess free CY1-4 was placed in pH 1.2 solution, pH 6.8 solution and deionized water, and shaken at 37 °C water bath for 48 h. The supernatants were filtered after centrifugation at 10000 rpm for 5 min, and then the filtrates were collected. The CY1-4 was measured by HPLC to calculate the solubility of CY1-4.

1.4. Prescription screening of CY1-4 nano-skeleton drug delivery system

The CY1-4 nano-skeleton drug delivery system was prepared by solvent evaporation method. CY1-4 was dissolved in the mixed solution of dichloromethane and anhydrous methanol (dichloromethane:anhydrous methanol = 3:1, *v* / *v*). Sylysia was dispersed in dichloromethane, HPMC was dispersed in dichloromethane, and DSPE-PEG_{2000} was dissolved in dichloromethane.

1.4.1. Preparation of CY1-4:Sylysia = 1:3 (*w* / *w*)

The CY1-4 solution was dropped into Sylysia dispersion liquid under magnetic stirring, and then sonicated for 30 min. After that, the CY1-4-Sylysia mixture was stirred for 24 h, and then evaporated to dryness under reduced pressure at 40 °C.

1.4.2. Preparation of CY1-4:Sylysia:HPMC = 1:3:1, 1:3:2 and 1:3:3 (*w* / *w* / *w*)

The CY1-4 solution was dropped into Sylysia dispersion liquid under magnetic stirring, and then sonicated for 30 min. After that, the HPMC dispersion liquid was dropped into the CY1-4-Sylysia mixture, stirred for 24 h, and evaporated to dryness under reduced pressure at 40 °C.

1.4.3. Preparation of CY1-4:Sylysia:HPMC:DSPE-PEG_{2000} = 1:3:3:3 (*w* / *w* / *w* / *w*)

The CY1-4 solution was dropped into Sylysia dispersion liquid under magnetic stirring, and then sonicated for 30 min. After that, the HPMC dispersion liquid was dropped into the CY1-4-Sylysia mixture, stirred for 24 h, and evaporated to dryness under reduced pressure at 40 °C. Subsequently, the DSPE-PEG_{2000} solution was added, ultrasonicated for 15 min, and evaporated to dryness under reduced pressure at 40 °C.

1.4.4. Determination of the solubility of CY1-4 in the prescription of CY1-4 nano-skeleton drug delivery system

The nano-skeleton drug delivery system with different prescriptions containing excess CY1-4 were placed in deionized water, and were shaken at 37 °C water bath for 48 h. The supernatant was filtered after centrifugation at 10000 rpm, the filtrate was collected, and the CY1-4 in each prescription was measured by HPLC to calculate the solubility of CY1-4.

1.5. Preparation of physical mixtures

CY1-4, Sylysia, HPMC and DSPE-PEG_{2000} were simply mixed according to the ratio of CY1-4:Sylysia:HPMC:DSPE-PEG_{2000} = 1:3:3:3 (*w* / *w* / *w* / *w*) until the uniform mixtures were obtained, which was the physical mixtures.

1.6. Characterization of MSNM@CY1-4

1.6.1. Scanning electron microscope (SEM)

CY1-4, Sylysia and MSNM@CY1-4 were respectively dispersed in the solvent, dropped on the copper net, dried in air, sprayed with gold to make them conductive, and observed by scanning electron microscope (MERLIN Compact) respectively.

1.6.2. Powder X-ray diffraction (PXRD)

The proper sample powders of CY1-4, Sylysia, HPMC, DSPE-PEG_{2000}, MSNM@CY1-4, Physical mixtures were placed on D/MAX-2400 rotating target anode X-ray diffractometer for determination. The crystallization of CY1-4, Sylysia, HPMC, DSPE-PEG_{2000}, MSNM@CY1-4, Physical mixtures were investigated respectively. X-ray generator: Cu; X-ray optical system; Flat graphite monochromator; Starting angle: 3.0 ° (2θ), ending angle: 50 ° (2θ); Speed: 5 ° / min.

1.6.3. Analysis of specific surface area and pore volume

Sylysia and MSNM@CY1-4 samples were degassed under vacuum and reduced pressure at 70 °C for 12 h, and then the proper Sylysia and MSNM@CY1-4 were measured using ASAP 2020 rapid specific surface and pore size distribution tester to analyze the specific surface area and surface porosity of Sylysia and MSNM@CY1-4. The specific surface area and pore volume of Sylysia and MSNM@CY1-4 were calculated by Brunauer-Emmett-Teller (BET) method and Barrett-Joyner-Halenda (BJH) method, respectively.

2. Supplementary Results

2.1. Maximum absorption wavelength of CY1-4

The A-λ light absorption curve of CY1-4 in the 190-600 nm spectral region is shown in Figure S1. The maximum absorption wavelength of CY1-4 was 258 nm, which could be determined that the detection wavelength of CY1-4 was 258 nm.

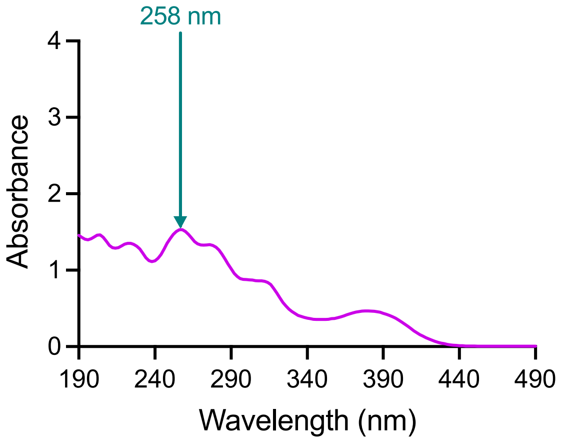

Figure S1. UV-vis spectra of CY1-4.

2.2. Solubility of CY1-4

The determination results of the solubility of CY1-4 are shown in Table S1. The solubility of CY1-4 in pH 1.2, pH 6.8 and deionized water was less than 0.1 mg / mL, which was an insoluble drug, and the solubility of CY1-4 had no obvious pH dependence.

Table S1. The solubility of CY1-4.

	**Solubility (μg / mL)**
Deionized water	1.16 ± 0.08
pH = 1.2	1.36 ± 0.04
pH = 6.8	1.19 ± 0.09

2.3. Prescription screening

Mesoporous silica nano-skeleton carrier material Sylysia, hydrophilic polymer material HPMC and amphiphilic lipid material DSPE-PEG$_{2000}$ were selected to construct various nano-skeleton systems, and the optimal formulation was screened to improve the solubility of CY1-4. All the synthesized nano-skeleton drug delivery systems were yellow-green solids, and the solubility of CY1-4 in the nano-skeleton drug delivery system are shown in Table S2. The solubility of CY1-4 was 1.16 μg / mL, and the solubility of CY1-4 in CY1-4-Sylysia nano-skeleton drug delivery system was 25.47 μg / mL, which was about 22.0-fold than that in CY1-4. The solubility of CY1-4 in CY1-4-Sylysia-HPMC nano-skeleton drug delivery system was 38.41-72.43 μg / mL, which was 33.1-62.4-fold than that in CY1-4. With the increase of HPMC content, the solubility of CY1-4 also increased. The solubility of CY1-4 in CY1-4-Sylysia-HPMC-DSPE-PEG$_{2000}$ nano-skeleton drug delivery system was 233.22 μg / mL, which was 201.1-fold than that in CY1-4, which was significantly higher than that of CY1-4, CY1-4-Sylysia nano-skeleton drug delivery system and CY1-4-Sylysia-HPMC nano-skeleton drug delivery system. Therefore, we screened out the best solubilization effect of CY1-4-Sylysia-HPMC-DSPE-PEG$_{2000}$ nano-skeleton drug delivery system. CY1-4:Sylysia:HPMC:DSPE-PEG$_{2000}$ = 1:3:3:3 (*w* / *w* / *w* / *w*) was the best prescription, which was named CY1-4 nano-skeleton drug delivery system (MSNM@CY1-4). The subsequent in vitro and in vivo studies will be conducted on MSNM@CY1-4.

Table S2. The solubility of MSNM@CY1-4.

	Solubility (μg / mL)
CY1-4:Sylysia = 1:3	25.47 ± 0.67
CY1-4:Sylysia:HPMC = 1:3:1	38.41 ± 1.94
CY1-4:Sylysia:HPMC = 1:3:2	52.32 ± 1.17
CY1-4:Sylysia:HPMC = 1:3:3	72.43 ± 1.27
CY1-4:Sylysia:HPMC:DSPE-PEG$_{2000}$ = 1:3:3:3	233.22 ± 8.94

2.4. Characterization of MSNM@CY1-4

2.4.1. Scanning electron microscope (SEM)

The scanning electron microscope (SEM) images of CY1-4, Sylysia and MSNM@CY1-4 are shown in Figure S2. The results showed that CY1-4 was existed in the form of irregular crystal, while no CY1-4 crystal was found in MSNM@CY1-4.

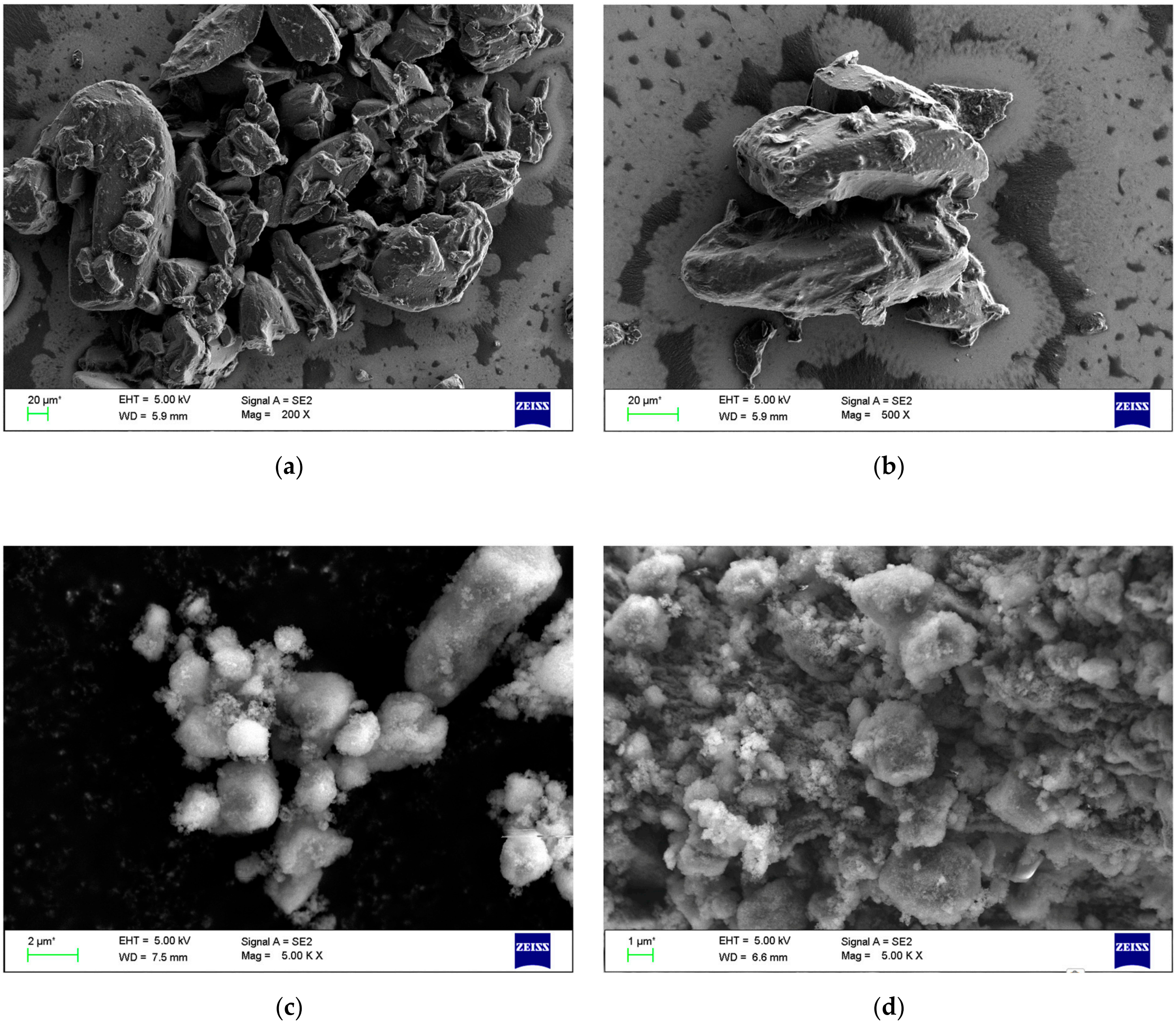

Figure S2. Topical SEM images of (**a**) pure CY1-4 and (**b**) local amplification of pure CY1-4, (**c**) Sylysia and (**d**) MSNM@CY1-4.

2.4.2. Powder X-ray diffraction (PXRD)

The powder X-ray diffraction results of MSNM@CY1-4 are shown in Figure S3. The results showed that CY1-4 exhibited crystal diffraction peaks at 6.55 °, 13.15 ° and 28.43 °,

indicating that CY1-4 was existed in a crystalline state. Sylysia and HPMC had no obvious crystallization peaks. DSPE-PEG2000 had two crystallization peaks at 19.17 ° and 23.44 °. The crystal peaks of CY1-4 still existed in the physical mixtures, while the crystall peaks of CY1-4 disappeared in MSNM@CY1-4, indicating that after CY1-4 and Sylysia, HPMC, DSPE-PEG2000 were prepared into MSNM@CY1-4 in the ratio of 1:3:3:3, CY1-4 was existed in the amorphous state.

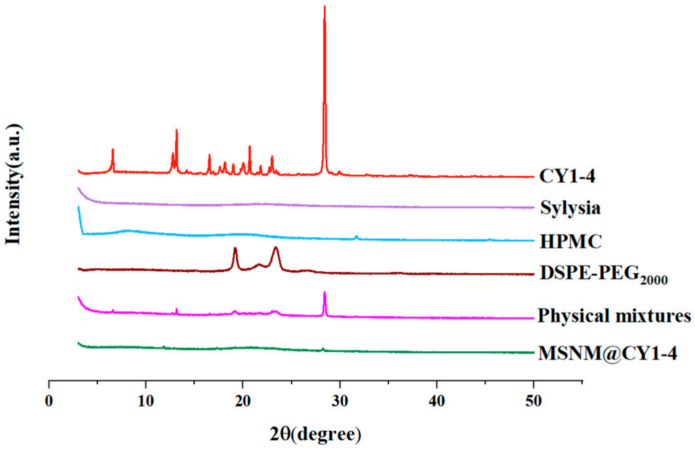

Figure S3. PXRD patterns of pure CY1-4, Sylysia, HPMC, DSPE-PEG2000, Physical mixtures and MSNM@CY1-4.

2.4.3. Analysis of specific surface area and pore volume

The specific surface area and pore volume of Sylysia and MSNM@CY1-4 were calculated by BET and BJH methods. The results are shown in Figure S4 and Table S3. The surface area of Sylysia was about 23.9-fold than that of MSNM@CY1-4, and the pore volume of Sylysia was about 23.9-fold than that of MSNM@CY1-4, indicating that CY1-4 successfully entered the nanopores of Sylysia.

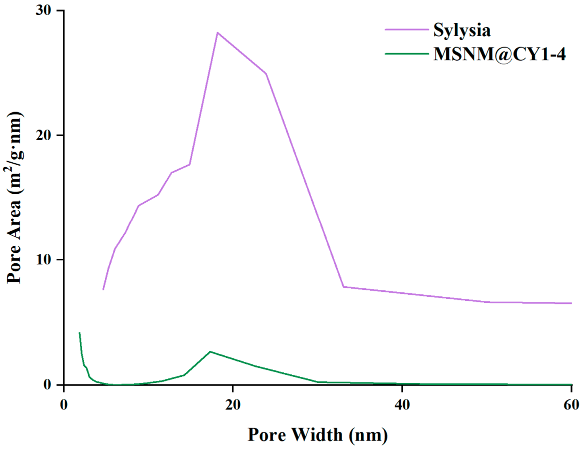

Figure S4. The BJH pore size distribution curves of Sylysia and MSNM@CY1-4.

Table S3. Specific surface areas and pore volumes of Sylysia and MSNM@CY1-4.

	BJH desorption cumulative surface area of pores (m^2 / g)	Pore Volume (cm^3 / g)
Sylysia	374.00	1.91
MSNM@CY1-4	33.92	0.18
